# GFP-Based Fluorescence Assay for CAG Repeat Instability in Cultured Human Cells

**DOI:** 10.1371/journal.pone.0113952

**Published:** 2014-11-25

**Authors:** Beatriz A. Santillan, Christopher Moye, David Mittelman, John H. Wilson

**Affiliations:** 1 Verna and Marrs McLean Department of Biochemistry and Molecular Biology, Baylor College of Medicine, Houston, Texas, United States of America; 2 Department of Molecular and Human Genetics, Baylor College of Medicine, Houston, Texas, United States of America; 3 Virginia Bioinformatics Institute, Virginia Tech, Blacksburg, Virginia, United States of America; 4 Department of Biological Sciences, Virginia Tech, Blacksburg, Virginia, United States of America; INSERM UMR S_910, France

## Abstract

Trinucleotide repeats can be highly unstable, mutating far more frequently than point mutations. Repeats typically mutate by addition or loss of units of the repeat. CAG repeat expansions in humans trigger neurological diseases that include myotonic dystrophy, Huntington disease, and several spinocerebellar ataxias. In human cells, diverse mechanisms promote CAG repeat instability, and in mice, the mechanisms of instability are varied and tissue-dependent. Dissection of mechanistic complexity and discovery of potential therapeutics necessitates quantitative and scalable screens for repeat mutation. We describe a GFP-based assay for screening modifiers of CAG repeat instability in human cells. The assay exploits an engineered intronic CAG repeat tract that interferes with expression of an inducible GFP minigene. Like the phenotypes of many trinucleotide repeat disorders, we find that GFP function is impaired by repeat expansion, in a length-dependent manner. The intensity of fluorescence varies inversely with repeat length, allowing estimates of repeat tract changes in live cells. We validate the assay using transcription through the repeat and engineered CAG-specific nucleases, which have previously been reported to induce CAG repeat instability. The assay is relatively fast and should be adaptable to large-scale screens of chemical and shRNA libraries.

## Introduction

Expansions of CAG trinucleotide repeats (TNRs) cause several neurological diseases in humans, including Huntington disease, myotonic dystrophy type 1, and a number of spinocerebellar ataxias [1], [2]. Long CAG tracts are unstable during transmission between generations, giving rise to progeny with additional CAG units (expansions) or with fewer units (contractions), but usually with a bias toward expansions. Expansions in the germ line lead to earlier disease onset and increased severity in affected individuals [1], while expansions in specific neurons exacerbate disease symptoms [3], [4]. Reducing repeat expansions or promoting repeat contractions—even partial contractions—would significantly advance therapy for TNR disorders.

One obstacle to devising therapies for shrinking expanded CAG repeats is the diversity of pathways that destabilize repeat tracts. Studies in model organism have identified a broad spectrum of DNA transactions—replication, recombination, DNA repair, and transcription, to name a few—that can contribute to TNR instability [5], [6], [7], [8]. Virtually any protein or process that exposes single DNA strands in CAG repeat regions allows formation of hairpins and slipped duplexes, which trigger repeat instability [9], [10]. In addition, studies in mice have revealed that mechanisms of TNR instability differ from tissue to tissue [11], [12], [13], [14], [15].

Investigations into TNR instability depend on methods to assess repeat variation. Traditionally, small-pool PCR [16] and GeneScan [17], [18] have provided effective tools for assessing TNR instability, but these labor-intensive methods do not scale well. Emerging methods such as Illumina and PacBio sequencing are promising because they enable high-throughput and precise measurement of repeat length changes [19], [20], [21]; however, they are still cost prohibitive for large-scale screens. Selection assays in yeast [22], [23], [24], [25], [26] and mammalian cells [27], [28], [29], [30], [31] offer powerful methods for testing cellular processes and candidate genes for their effects on TNR instability. But the frequency of detected events in these assays is generally too low for high-throughput screens.

Here, we described a fast and scalable GFP-based fluorescence assay for assessment of CAG repeat instability. Like our selection assays in human cells, this fluorescence assay is based on the ability of long CAG tracts in an intron to interfere with gene expression. Importantly, the degree of fluorescence depends in an inverse manner on the length of the repeat tract, providing a noninvasive estimate of the length of a CAG repeat tract in living cells.

## Materials and Methods

### Plasmids

The GFP-Pem1 plasmid, a kind gift from Dr. Lei Li, was created by the insertion of portions of the large intron in the *Rattus norvegicus Rhox5* gene into the eGFP gene on the pEGFP-N1 backbone (Clontech Laboratories, Mountain View, CA). The resulting GFP minigene contains an efficiently spliced intron approximately 1.5 kb in length. The plasmid was further modified to contain a polylinker in the intron [32]. We inserted a (CAG)_89_ repeat tract, along with 129 bp of flanking sequence, into the NotI site in the polylinker to generate plasmid pGFP-Pem1-CAG_89_. The CAG repeat tract, which was originally cloned from a myotonic dystrophy patient [33], retained 43 nucleotides 5′ and 19 nucleotides 3′ of human sequences flanking the repeat tract at the myotonic dystrophy locus.

**Figure 1:**
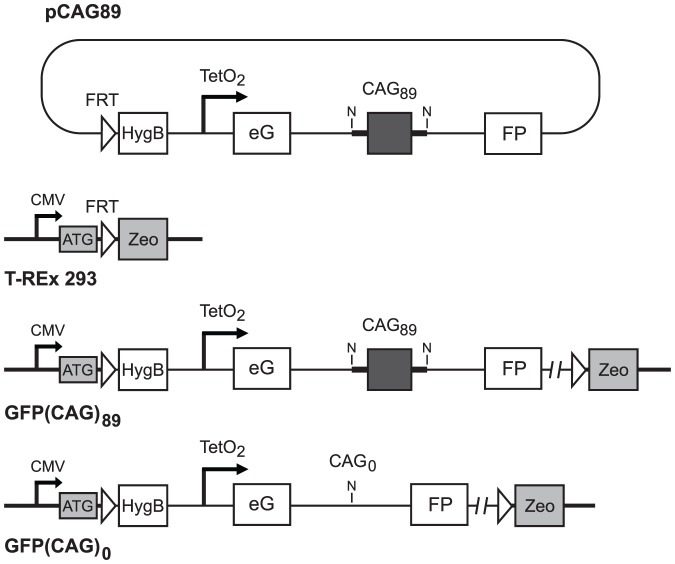
Construction of GFP(CAG)_89_ and GFP(CAG)_0_ cell lines. The GFP(CAG)_89_ cell line was generated by FLP-mediated site-specific recombination between the FRT sites in plasmid pCAG89 and in the genome of T-REx 293 cells, creating a chromosomal integrant. The GFP(CAG)_0_ cell line was generated in a similar way using plasmid pCAG0, which was derived from pCAG89 by cleavage with NotI (N) and religation, a procedure that eliminates the CAG repeat along with the flanking human sequences. HygB is hygromycin B; Zeo is zeocin.

To insert the modified GFP gene into the pcDNA5/FRT/TO vector (Invitrogen, Carlsbad, CA), which carries the inducible CMV/TetO_2_ hybrid promoter (cytomegalovirus immediate early—CMV—promoter plus two tetracycline operator 2—TetO_2_—sites) and an FRT site for insertion in T-REx HEK293 cells, we first introduced an XhoI linker at the MfeI restriction in pGFP-Pem1-CAG vector. We digested pGFP-Pem1-CAG-XhoI with PspOMI and XhoI, isolated the modified GFP gene, which was then inserted into compatible NotI and XhoI sites in the polylinker in pcDNA5/FRT/TO, generating plasmid pCAG89 (Figure 1). To create plasmid pCAG0, we digested pCAG89 with NotI, which removes the CAG repeat tract and 129 nucleotides of flanking DNA, and recircularized the backbone.

### Cell Lines

To create human cells with chromosomally integrated targets, we co-transfected the Flp recombinase-encoding vector, pOG44, with the pCAG89 or the pCAG0 vector into the Flp-In T-REx 293 Cell Line (Invitrogen, Carlsbad, CA), using Lipofectamine 2000 Transfection Reagent (Invitrogen) (Figure 1). The cells were maintained in DMEM medium supplemented with 10% FBS at 37°C in 5% CO_2_ for two days. The cells were then trypsinized, diluted, and re-plated for colony formation. Individual colonies were screened for appropriate drug sensitivity. FLP-recombinase-mediated insertion of the pCAG89 and pCAG0 plasmids should confer resistance to 200 µg/mL hygromycin B (Invitrogen, Carlsbad, CA) and sensitivity to 100 µg/mL zeocin (Invitrogen). Resistance to blasticidin verified that the isolated clones retained the gene for the Tet Repressor, which controls the doxycycline-inducible TetO_2_ CMV promoter. Individual colonies were sequenced to determine the length of the CAG repeat tract in the chromosomal GFP minigene. One cell line with 89 CAG repeat units—designated GFP(CAG)_89_—and one cell line with 0 CAG repeats—designated GFP(CAG)_0_—were used in the experiments described here. The GFP(CAG)_0_ cell line serves as a control to identify the maximum level of GFP expression.

All cell lines were maintained in plates containing DMEM GIBCO 4.5 g/L D-Glucose; L-Glutamine (GIBCO, Grand Island, NY) supplemented with 10% HyClone Fetal Bovine Serum, Standard (Thermo Scientific, Logan, UT) in a humidified incubator at 37°C with 5% CO_2_.

### Flow Cytometry

We performed flow cytometry analyses using the BD LSRFortessa Cell Analyzer (BD Biosciences, San Jose, CA). All data were analyzed with BD FACSDiva Software 6.1.2 (BD Biosciences). Prior to cytometry analysis, cells were trypsinized and diluted to 2×10^6^ cells/mL in complete medium. We filtered cell solutions in 35 µm cell strainer cap tubes (BD Biosciences) and kept them on ice until analysis. For each sample, eGFP fluorescence was analyzed with a 488 nm wavelength blue laser. Because all cells with a GFP gene show some degree of fluorescence, we used gates to define the fluorescent population of interest. When we were interested in a subpopulation that was more fluorescent that the main population, we arbitrarily defined those cells that passed the appropriate gate as GFP+ cells. For transcription-induced repeat instability, we used the distribution of GFP(CAG)_0_ cells, fully induced with 2 µg/mL doxycycline for 24 hours, to define the gates for GFP+ cells. Frequencies of GFP+ cells were calculated as the number of GFP+ cells divided by the total number of cells counted.

Fluorescence-activated cell sorting was carried out on a BD FACSAria II Cell Sorter (BD Biosciences). We prepared cells identically for flow cytometry and cell sorting. The catch media was 50% serum and 50% complete medium, and the samples were kept at 4°C throughout sorting. Individual cells were sorted into individual wells of 96-well plates. The surviving cells—typically 10–20%—were grown into populations, and their repeat tracts were sequenced.

### Analysis of CAG Tract Lengths

We determined CAG tract lengths by PCR amplification and sequence analysis. PCR mixtures consisted of 1 µL DNA, 0.3 mM dNTPs, 1.75 mM MgCl_2_, 1.2 mM betaine (Sigma-Aldrich, St. Louis, MO), 1 U ChromaTaq DNA Polymerase (Denville Scientific Inc., Denville, NJ), 4 µL 5× ChromaTaq Buffer, and dH_2_O to 20 µL. PCR mixtures contained 0.5 µM each forward (5′-AAGAGCTTCCCTTTACACAACG) and reverse (5′-TACCAGGACAGCAGTGGTCA) primer, which are located on either side of the repeat tract, about 250 nucleotides away. The PCR program consisted of 2 minutes at 94°C, followed by 39 cycles of 94°C for 15 seconds, 60°C for 30 seconds, and 72°C for 45 seconds, with a final extension cycle at 72°C for 10 minutes. PCR products were run on a 1.5% agarose gel and visualized with ethidium bromide. For sequence analysis, PCR products were isolated by either a QIAprep Spin Miniprep Kit (QIAGEN, Hilden, Germany) or a QIAquick Gel Extraction Kit (QIAGEN). Samples (100 ng) were sequenced at Lone Star Labs (Houston, Texas). To determine repeat length, AB Sequence Scanner Software v1.0 (Applied Biosystems, Foster City, CA) was used to analyze chromatographs of the sequencing reactions.

### Quantitative Reverse Transcription PCR

We extracted RNA from trypsinized cell samples resuspended in PBS, using the RNeasy Mini Kit (QIAGEN, Hilden, Germany) supplemented with β-mercaptoethanol (Sigma-Aldrich), according to the manufacturer's protocol. First strand synthesis was performed on 1 µg RNA per sample in a final volume of 20 µL, using the iScript cDNA Synthesis Kit (Bio-Rad Laboratories, Hercules, CA), following the manufacturer's instructions. We prepared the quantitative PCR reaction using the QuantiFast SYBR Green PCR Kit (QIAGEN) and 2 µL cDNA reaction per sample in a final volume of 25 µL, according to the manufacturer's protocol. PCR reactions contained 0.5 µM of each primer. Primer set 1 (5′-CAGAAGAACGGCATCAAGGT and 5′-CTGGGTGCTCAGGTAGTGGT) and primer set 2 (5′-TATATCATGGCCGACAAGCA and 5′-GGGTGTTCTGCTGGTAGTGG) each amplified segments of GFP exon 2. As designed, each primer set should amplify equally well both the correct GFP spliced product and the aberrant spliced product that includes the CAG repeat tract. The amplifications were performed on a CFX96 Real-Time PCR Detection System (Bio-Rad Laboratories). The PCR program consisted of 5 minutes at 95°C, followed by 40 cycles of 95°C for 10 seconds and 60°C for 30 seconds. Following amplification, the temperature was ramped from 60°C to 95°C at a rate of 0.5°C every 5 seconds for a melt curve analysis. All samples were analyzed in duplicate and normalized to β-actin. Gene expression was determined by the ΔΔC_t_ method [34].

### Northern Blot

We generated a probe for EGFP mRNA by PCR-amplifying the complete EGFP gene in pEGFP-N1. The probe was radiolabeled using the DECAprime II Kit (Ambion, Austin, TX) and ^32^P-dCTP, according to the manufacturer's instructions. RNA was extracted from cultured cells with the RNeasy Mini Kit (QIAGEN) and subjected to electrophoresis on a 1% agarose denaturing formaldehyde gel (2.2 M formaldehyde; 200 mM MOPS, pH 7.0; 50 mM sodium acetate; 10 mM EDTA). The products were then transferred to a Amersham Hybond N+ nylon membrane (GE Healthcare, Piscataway, NJ) overnight and hybridized with the labeled probe, using ULTRAhyb Ultrasensitive Hybridization Buffer (Ambion).

## Results

### A GFP-Based Fluorescence Assay for CAG Repeat Instability

To develop a rapid assay for CAG repeat instability, one with a potential for rapid screening of drugs and candidate genes, we tested the effects of a long CAG repeat on expression of a chromosomal copy of a GFP minigene. In previous studies using the APRT gene and the HPRT minigene, we found that placing a long CAG repeat in an intron reduced expression below levels required for cell survival under selective conditions, giving rise to a selective assay for repeat contraction [28], [29]. To determine whether long CAG tracts also affected expression of the GFP minigene, we inserted a CAG_89_ repeat tract into the intron in the minigene, placed the minigene under control of a Tet-On promoter, and deposited it in a chromosome by FLP-mediated recombination into the FRT site in the genome of T-REx HEK293 cells (Figure 1). In parallel to the GFP(CAG)_89_ cell line, we also generated a GFP(CAG)_0_ cell line, which carries the same GFP minigene at the same chromosomal location, but without a CAG repeat tract (Figure 1).

**Figure 2:**
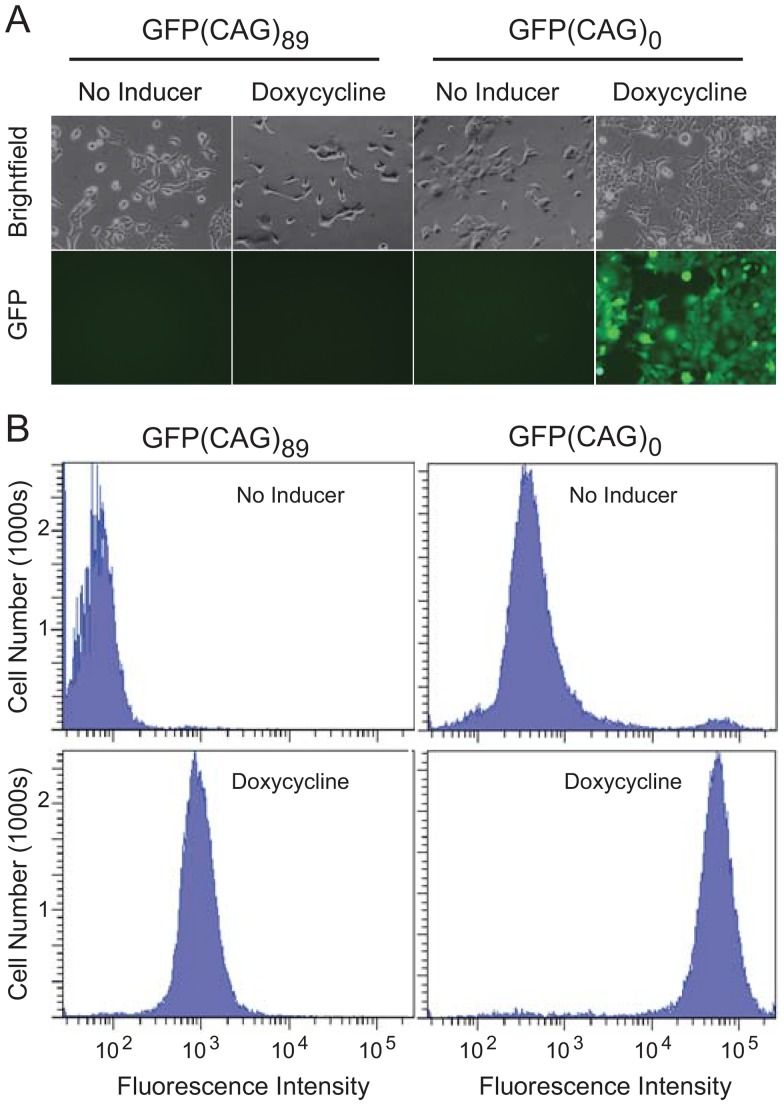
GFP fluorescence in GFP(CAG)_89_ cells and in GFP(CAG)_0_ cells before and after addition of doxycycline. **A**. Fluorescence microscopy. Brightfield and fluorescent images of the same cell populations are shown. **B**. Flow cytometry. In each cell line, with or without added doxycycline, 50,000 cells were analyzed by flow cytometry and plotted as a histogram. Although not shown, unmodified T-REx cells—in the presence or absence of doxycycline—give a distribution that is indistinguishable from uninduced GFP(CAG)_89_ cells.

To test inducibility of expression from the Tet-On promoter, we grew both cell lines in the presence or absence of doxycycline for two days and then observed them by microscopy (Figure 2A). As expected, neither cell line exhibited visible fluorescence in the absence of doxycycline. Upon addition of doxycycline, GFP(CAG)_0_ cells fluoresced intensely, indicating that the Tet-On system is functional and that the repeat-containing intron is correctly spliced. By contrast, GFP(CAG)_89_ cells did not reveal visible GFP fluorescence in the presence of doxycycline. Given that both cell lines contain the GFP gene in the same genomic location, we conclude that the lack of fluorescence in GFP(CAG)_89_ cells is due to the presence of the repeat tract.

To observe the distribution of GFP fluorescence in the cell populations, both cell lines were analyzed by flow cytometry. Without induction—with basal transcription only—GFP(CAG)_0_ cells fluoresced more intensely than the GFP(CAG)_89_ cells (Figure 2B). Addition of doxycycline increased the mean fluorescence intensities of both cell lines, with GFP(CAG)_0_ cells once again fluorescing more intensely than the GFP(CAG)_89_ cells, supporting the observations made by microscopy. In addition, given that the fluorescence intensities were higher in GFP(CAG)_0_ cells than in GFP(CAG)_89_ cells for both the induced and uninduced populations, these results further support the idea that the CAG_89_ repeat tract inhibits GFP expression. Importantly, the induced GFP(CAG)_0_ and GFP(CAG)_89_ cells formed discrete populations in histograms of fluorescence intensity.

In the GFP(CAG)_0_ cells, we noted the presence of a small population of highly fluorescent cells in the uninduced cell population, and also a small population of nonfluorescent cells in the induced population (Figure 2B). The highly fluorescent cells, which we will refer to as GFP+ cells, might arise by loss of the Tet repressor; the nonfluorescent cells could arise by loss of the GFP reporter gene. We tested those possibilities by preselecting cells with blasticidin, whose resistance gene is linked to the Tet repressor gene, and with hygromycin, whose resistance gene is linked to the GFP gene. Treatment with blasticidin reduced the background of GFP+ cells by 8-fold; treatment with hygromycin reduced the level of nonfluorescent cells by 4-fold. Thus, to increase the sensitivity of the assay, we routinely pre-treated cells with blasticidin and hygromicin.

**Figure 3:**
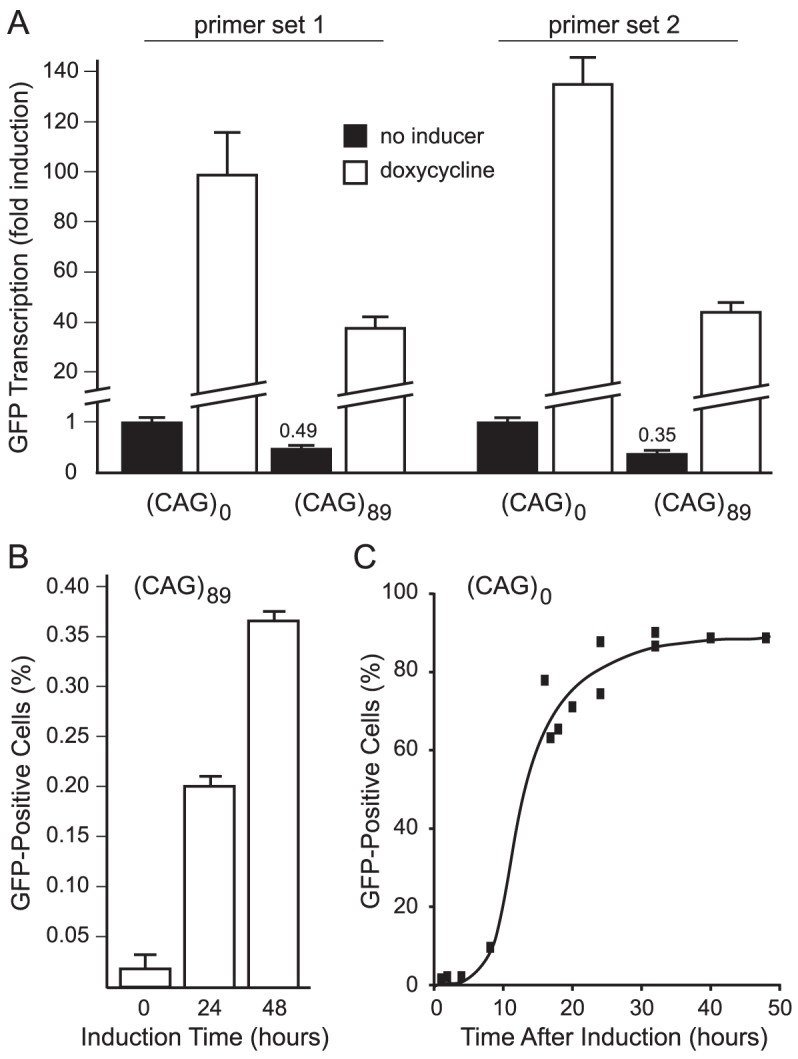
Doxycycline-induced transcription of the GFP gene. **A**. Quantification of doxycycline-induced GFP transcription in GFP(CAG)_89_ and GFP(CAG)_0_ cell lines. Cells were induced with doxycycline for three days prior to analysis. Two independent primer sets, both specific for GFP exon 2, were used to amplify GFP transcripts by quantitative RT-PCR. For each primer set, the individual results were internally normalized to β-actin and then to the levels of GFP transcripts in uninduced GFP(CAG)_0_ cells. The values for the uninduced levels of GFP transcripts in GFP(CAG)_89_ cells are indicated. The increase over uninduced levels in GFP(CAG)_89_ cells was 76-fold for primer set 1 and 98-fold for primer set 2. **B**. Transcription-induced changes in numbers of GFP+ cells in GFP(CAG)_89_ cells. Cells were treated with doxycycline for 0, 24, or 48 hours prior to analysis for GFP+ cells by flow cytometry, using the High gate indicated in Figure 4A to define the population of GFP+ cells. The frequencies of GFP+ cells at 0, 24, and 48 hours, respectively, were 0.02±0.013%, 0.20±0.02%, and 0.37±0.01%, as determined by counting three samples of 50,000 cells. C. Kinetics of induction of GFP expression in GFP(CAG)_0_ cells. Doxycycline (2 µg/mL) was added at time 0 to wells of 6-well plates containing 100,000 GFP(CAG)_0_ cells. Individual wells were harvested at the indicated times and analyzed by flow cytometry, using the High gate indicated in Figure 4A to define the population of GFP+ cells.

To quantify GFP expression, we analyzed induced and uninduced cells by quantitative RT-PCR, using two independent primer sets. As shown in Figure 3A, doxycycline induced transcription of the GFP gene by approximately 100-fold in both cell lines. Although fold inductions were indistinguishable in the two cell lines, the presence of the CAG_89_ repeat tract was consistently associated with a 2- to 3-fold reduction in transcript levels relative to levels in cells with the CAG_0_ repeat tract. In GFP(CAG)_89_ cells, induction of transcription increased the frequency of highly fluorescent cells—GFP+ cells—in a time-dependent manner (Figure 3B), as we demonstrated previously for transcription-induced HPRT+ cells in our HPRT selection assay [28].

### Fluorescence Intensity is Inversely Proportional to Repeat Length

**Figure 4:**
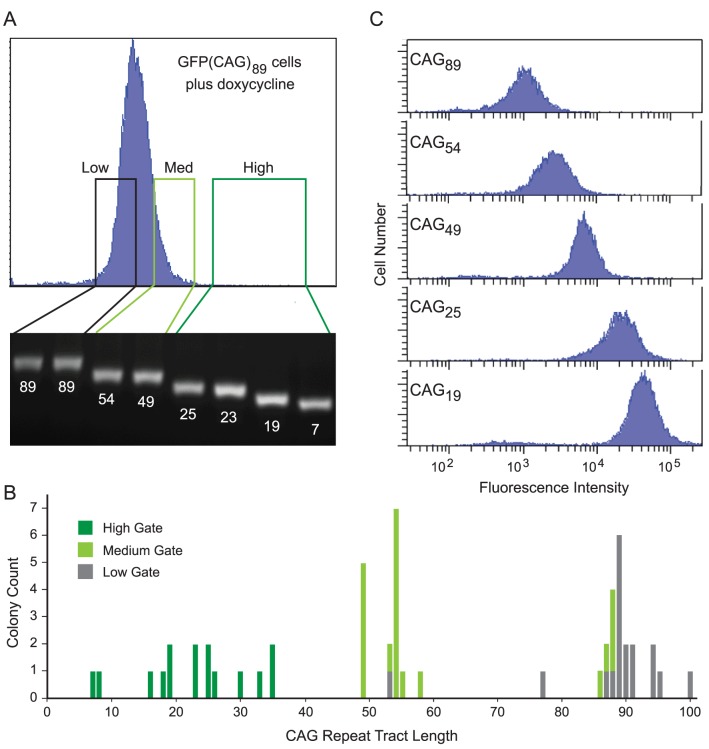
Relationship between GFP fluorescence intensity and CAG repeat tract length. **A**. Isolation of cells from different parts of the distribution of fluorescence intensity. Induced GFP(CAG)_89_ cells were sorted according to the indicated gates, single cells were grown into colonies, and their CAG tracts were amplified by PCR and sequenced. **B**. Distribution of tract lengths in cells sorted by fluorescence intensity. **C**. Fluorescence intensity of cells with different CAG tract lengths.

Because transcription induces repeat instability, as we previously showed, we expected that addition of doxycycline here would do the same, allowing us to test the relationship between repeat length and fluorescence intensity. We induced GFP(CAG)_89_ cells with doxycycline for three days to stimulate repeat instability, and then sorted cells from this distribution based on GFP fluorescence intensity. The High intensity gates corresponded to the gates used to measure transcription-induced repeat instability, while the Medium and Low gates corresponded to regions of lesser fluorescence intensity (Figure 4A). Individual colonies were grown and analyzed by DNA sequencing and by FACS.

Sequence analysis revealed that CAG tract length correlated inversely with the fluorescence intensity defined by the original gates (Figure 4B). Sorting through the High intensity gate yielded 15 colonies with repeats from 7 to 35 units in length; sorting through the Medium gate gave 15 colonies with repeats 49 to 58 units; and sorting through the Low gate yielded 17 colonies with repeats from 77 to 100 units. Notably, the Low gate yielded 4 clones with repeat tracts that were at least 5 units longer than the parental tract, including one that was 11 units longer. To verify the relationship between repeat length and fluorescence intensity, we grew several colonies into populations and analyzed them by flow cytometry. As shown in Figure 4C, there is a reasonable inverse correlation between CAG tract length and fluorescence intensity.

### Long Repeat Tracts Cause Aberrant Splicing

**Figure 5:**
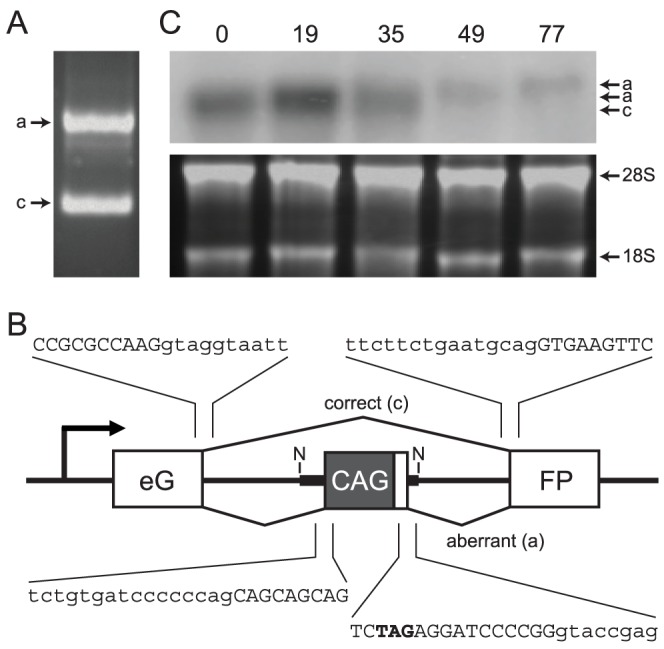
Aberrant splicing at the CAG repeat tract. **A**. Amplification of GFP transcripts from induced GFP(CAG)_89_ cells by RT-PCR. **B**. Splice junctions in normal and aberrantly spliced GFP mRNA. The lower band in A corresponds to correctly spliced GFP mRNA, with sequences around the splice sites indicated above the gene. The upper band in A corresponds to aberrantly spliced mRNA that included the CAG repeat as an extra exon. Splice sites at the ends of the CAG exon are indicated below the gene. In all cases, upper case letters were retained in the spliced product and lower case letter were eliminated. An in-frame stop codon is highlighted in bold. The Not1 site that was used to clone the CAG repeat tract, along with its 129 bp of flanking sequences, is indicated as N. **C**. Northern blot analysis of GFP transcripts in induced cell lines with different lengths of CAG repeat tracts. Numbers above the lanes indicate the length of the CAG repeat tract. The lane labeled “0” is from GFP(CAG)_0_ cells. The upper panel shows the Northern blot, using a hybridization probe made from the full-length EGFP gene. The lower panel shows rRNA bands present in the ethidium bromide-stained gel prior to transfer for blot analysis.

In our previous studies, we had showed that long CAG tracts interfered with correct splicing of the APRT and HPRT genes [28], [29]. To determine the effects of CAG repeat tracts on splicing of the GFP minigene, we induced transcription in GFP(CAG)_89_ cells and subjected the RNA to RT-PCR, using primers in the GFP exons. As shown in Figure 5A, we observed two major products. Sequencing revealed that the lower band corresponded to the correctly spliced GFP transcript, encoding functional GFP (Figure 5B). The upper band corresponded to an aberrantly spliced transcript that contained approximately 300 extra nucleotides between the two GFP exons. The extra exon contained the CAG repeat tract plus 38 extra nucleotides immediately downstream of the repeat tract (Figure 5B), which matches exactly with the aberrant splicing we observed in the ARPT and HRPT selection assays [29]. The CAG repeat likely behaves the same in these different genes because the signals responsible for aberrant splicing—the donor and acceptor splice sites and the CAG repeat itself—are contained within the Not1 fragment used to move the repeat tract from gene to gene (Figure 5B). As expected, the GFP(CAG)_0_ cells, which lack the entire Not1 fragment, yield only the correctly spliced product (Figure 5C).

The extra exon included in the aberrant splice product in GFP(CAG)_89_ cells renders the transcript incapable of making functional GFP because it contains an in-frame termination codon, highlighted in Figure 5B, and because it alters the reading frame of the downstream GFP exon. As shown in the Northern blot analysis in Figure 5C, the correctly spliced (short) transcript was not visible with longer-length repeat tracts. In addition, the amount of aberrantly spliced transcript tended to decrease with increasing length of the CAG tract. Both of these observations are consistent with our previous results [29]. Thus, long CAG repeat tracts decrease production of correct GFP mRNA, in part by promoting inclusion of the CAG tract in aberrantly spliced GFP mRNA and in part by decreasing overall transcription.

### Transcription Versus Other Treatments

Visualizing GFP fluorescence requires transcription of the GFP gene, yet transcription itself causes repeat instability (Figure 3B). For GFP fluorescence to be broadly useful as an assay for CAG repeat instability, it must be possible to detect the effects of other treatments above those induced by transcription. Because transcription-induced CAG contractions accumulate with time of transcription [28], one way to limit the background due to transcription is to reduce the time of transcription to the minimum required for robust GFP expression. To determine the kinetics of GFP expression, we added doxycycline to GFP(CAG)_0_ cells and measured the percentage of the population that expressed GFP over time. As shown in Figure 3C, expression of GFP reached a plateau at about 24 hours.

**Figure 6:**
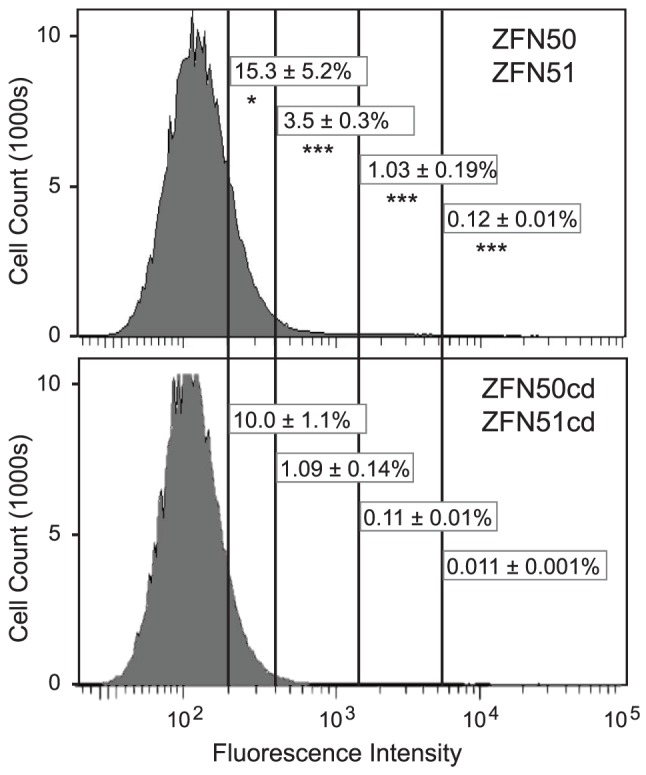
Stimulation of GFP+ cells by transfection with ZFN50/ZFN51. GFP(CAG)_89_ cells were transfected with 1.25 µg of each ZFN plasmid to a total of 2.5 µg per well of a 6-well plate. Cleavage-dead (cd) versions of ZFN50 and ZFN51, which carry the inactivating D450A mutation in the FokI cleavage domain and the R487D or the D483R obligate-heterodimer mutations in their dimerization domains, were used as transfection controls; they do not cleave CAG repeat tracts in vitro, nor do they stimulate CAG repeat contractions in GFP(CAG)_89_ cells [36]. The numbers presented in the figure show the mean and standard deviation from two experiments, each with three independent samples, for a total of six measurements for each ZFN. The numbers associated with each gate include all cells to the right of the gate. The numbers and gates used to derive them are displayed on a pair of histograms from parallel transfection samples for each ZFN. A total of 2.0×10^6^ cells were analyzed for ZFN50/ZFN51 transfections, and 2.2×10^6^ cells were analyzed for the cleavage-dead control ZFNs. Statistical significance was determined by a two tailed *t*-test of means of the corresponding gates of the active and inactive ZFNs (* = *P*<.05; *** = *P*<0.0001).

To determine whether we could see a stimulation of contractions over the background level of GFP+ cells induced by transcription, we treated GFP(CAG)_89_ cells with a zinc-finger nuclease (ZFN50/ZFN51) that cleaves CAG repeat tracts [35], [36]. We transfected ZFNs into cells and then after 72 hours added doxycycline for 24 hours to induce GFP expression. Gates were selected in the control population transfected with cleavage dead (cd) versions of the ZFNs to distinguish the highest 10%, 1%, 0.1%, and 0.01% of cells, according to their fluorescence intensity (Figure 6). Populations of cells transfected with ZFN50/ZFN51 showed a significant increase in GFP+ cells in every gate relative to control cells (Figure 6). In the two most stringent gates (0.1% and 0.01%), the active ZFN showed a 10-fold increase in GFP+ cells, a result that matches our previous experiments with our selective APRT and HRPT systems [35]. Characterization of the repeat tracts in isolated GFP+ cells showed that all carried modifications of the CAG repeat tract: 13 contained contractions and 1 contained a deletion that removed the repeat tract. These results confirm that it is possible to see effects on CAG repeat instability above the background of events due to transcription.

## Discussion

In this report, we describe a GFP-based fluorescence assay for analysis of the instability of CAG repeat tracts. The assay is based on the ability of long, intronic CAG repeat tracts to interfere with expression of the gene in which they reside. Importantly—and uniquely—this assay provides the first method for estimating the length of CAG repeat tracts in living cells.

The GFP-based fluorescence assay has several advantages over the selection assays we have used previously [28], [29], [31], [37], [38], [39], [40], [41]. First, the fluorescence assay is faster, taking just a few days instead of two to three weeks. Second, the fluorescence assay permits detection and analysis of a wider range of tract lengths, whereas our selection assays only detect cells that have fewer than 38 repeats. For example, Changes in the spectrum of events potentially can provide insights into the mechanism of instability induced by a treatment. Third, the absolute values for the frequencies of contractions—induced by transcription and ZFNs—are higher than for similar treatments in our selection assays, for reasons that are not entirely clear. For example, ZFN treatments in our selection assay gave frequencies of 0.01%, whereas those same treatments in the fluorescence assay gave frequencies above 1%. Similarly, transcription in our selective system gave frequencies around 0.001%, whereas in the fluorescence assay it gave frequencies of 0.2%. The higher frequencies make the fluorescence assay amenable to high-throughput protocols for screening chemical and shRNA libraries for effects of CAG repeat instability. Thus, the GFP-based fluorescence assay represents a significant advance over our previous selection assays for investigating CAG repeat instability, combining the speed and flexibility of cell culture with the screening power of flow cytometry.

One surprising and useful feature of this fluorescence assay is that GFP expression is inversely dependent on the length of the CAG repeat tract. The relationship between tract length and GFP expression means that the assay can be used to test a broad spectrum of changes to the repeat tract, potentially including expansions as well as contractions. This property of the system is likely a combination of factors. As we discussed previously [29], the CAG tract may behave as an exonic splicing enhancer (ESE), mimicking the CA-rich ESE motifs that function to promote splicing in vivo and in vitro [42], [43], [44], [45], [46], [47]. Because the 5′ splice site immediately downstream of the CAG tract is fairly weak (ESEfinder score of 4.4 [42]), its function may depend on the presence of the ESE, with longer CAG tracts promoting more efficient use of the splicing signal [29]. A second contributing factor may be that CAG repeat tracts interfere with transcription in a length-dependent manner. We have shown that a CAG_89_ tract decreases transcript levels by 2- to 3-fold (Figure 3). Supporting this idea, several in vitro studies have shown that transcription stalls at DNA sequences such as CAG tracts that can form secondary structures [48], [49], [50], [51], [52], [53], [54]. Third, the presence of a premature termination codon in the “CAG exon” may induce nonsense-mediated decay, although its position near the splice junction may limit its effectiveness [55]. Finally, it is possible that CAG repeats may induce heterochromatin-mediated silencing of the GFP gene in a length-dependent manner, as has been observed in other systems [56]. Regardless of the mechanism, fluorescence intensity, by serving as a reporter for the length of the repeat, provides a powerful tool for elucidating mechanisms of repeat instability.

This fluorescence-based assay has a potential limitation. As described here, the activity of the GFP gene must pass a threshold of expression for GFP+ cells to be detected by flow cytometry. As shown here, cells can become GFP+ by contraction of the CAG repeat tract (Figure 4B). In principle, however, treatments that increased expression of the GFP reporter gene—by increasing transcription or by stabilizing either the mRNA or protein—could increase the number of GFP+ cells, without increasing the number of cells carrying contractions of the CAG repeat tract. This eventuality would show up during analysis of the repeat tracts in GFP+ cells.

In this report, we have focused on the use of the fluorescence assay to detect repeat contractions. As we have discussed previously in relation to our selection systems, contractions in human cells are good predictors of processes that influence repeat instability in model organisms and, by extension, in human patients [39]. The fluorescence assay, however, offers the possibility of directly detecting CAG repeat expansions. Repeat lengths in the range of 50 to 60 CAG repeat units should provide sufficient fluorescence intensity that decreases in intensity caused by expansions of the repeat can be detected. In addition, because both contractions and expansions will be visible in the same assay, treatments that cause differential effects on contractions and expansions will be immediately apparent. Processes that cause a bias toward expansions may be more relevant to the disease processes in patients, which display a strong bias toward expansions in germ line and somatic tissues. By contrast, treatments that cause a strong bias toward contractions may provide clues to treatments designed to shrink repeat tracts in patients.
